# Protein Defense Systems against the Lantibiotic Nisin: Function of the Immunity Protein NisI and the Resistance Protein NSR

**DOI:** 10.3389/fmicb.2016.00504

**Published:** 2016-04-12

**Authors:** Sakshi Khosa, Marcel Lagedroste, Sander H. J. Smits

**Affiliations:** Lantibiotic Immunity and Resistance, Institute of Biochemistry, Heinrich-Heine-UniversityDuesseldorf, Germany

**Keywords:** lantibiotics, nisin, resistance, immunity, human pathogen, antimicrobial peptides

## Abstract

Lantibiotics are potential alternatives to antibiotics because of their broad-range killing spectrum. The producer strain is immune against its own synthesized lantibiotic via the expression of two proteins LanI and LanFEG. Recently, gene operons are found in mainly human pathogenic strains, which confer resistance against lantibiotics. Of all the lantibiotics discovered till date, nisin produced by some *Lactococcus lactis* strains is the most prominent member. Nisin has multiple mode of actions of which binding to the cell wall precursor lipid II and subsequent insertion into the bacterial membrane to form pores are the most effective. The nisin producing strains express the lipoprotein NisI to prevent a suicidal effect. NisI binds nisin, inducing a reversible cell clustering to prevent nisin from reaching the membrane. Importantly NisI does not modify nisin and releases it as soon as the concentration in the media drops below a certain level. The human pathogen *Streptococcus agalactiae* is naturally resistant against nisin by expressing a resistance protein called *Sa*NSR, which is a nisin degrading enzyme. By cleaving off the last six amino acids of nisin, its effectiveness is 100-fold reduced. This cleavage reaction appears to be specific for nisin since *Sa*NSR recognizes the C-terminal located lanthionine rings. Recently, the structures of both NisI and *Sa*NSR were determined by NMR and X-ray crystallography, respectively. Furthermore, for both proteins the binding site for nisin was determined. Within this review, the structures of both proteins and their different defense mechanisms are described.

## Introduction

Since the beginning of the last century, the heterogeneous group of bacteriocins have become an interesting research topic for various applications like food preservatives or pharmaceutical purposes as antibiotic alternatives (Cleveland et al., 2001; Cotter et al., 2012). Bacteriocins are small, ribosomally synthesized peptides of which some possess high antimicrobial activity (Tagg et al., 1976; Cotter et al., 2005b).

Within the group of bacteriocins a large family exists, which are called lantibiotics (Jung, 1991; Willey and van der Donk, 2007; Bierbaum and Sahl, 2009; Alvarez-Sieiro et al., 2016). These are small antimicrobial peptides, which are post-translationally modified and contain uncommon amino acids (Deyhydrobutyrine or Dehydroalanine). The linkage of these with cysteine residues results in the formation of characteristic thioether bridges, called lanthionine rings. These lanthionine rings ensure the high antimicrobial activity against various bacteria. Lantibiotics are highly potent as depicted by the observation that already nanomolar concentrations are sufficient to fulfill their antimicrobial activity (Delves-Broughton et al., 1996; Chatterjee et al., 2005). Furthermore, the lanthionine rings ensure that lantibiotics are intrinsically resistant against proteolysis by unspecific degrading enzymes.

Active lantibiotics are able to inhibit the growth of Gram-positive as well as Gram-negative bacteria. Some exhibit multiple modes of action, of which binding to lipid II, thereby inhibiting cell wall synthesis, and pore formation are the most prominent ones (Héchard and Sahl, 2002; Bierbaum and Sahl, 2009). Due to the high activitiy in combination with the high stability lantibiotics are considered as usefull compounds for medical treatment and food preservatives. One example is nisin produced by some *Lactococcus lactis* strains which is linked to the potential biomedical application against bacterial mastitis, treatment of methicillin-resistant *Staphylococcus aureus* (MRSA) and enterococcal infections. Other examples are the lantibiotics Gallidermin/Epidermin, which are associated with acne, eczema, follicultis, impetigo as possible compound for treatment (Cotter et al., 2005a).

Within the lantibiotic producer strains, the structural genes for the lantibiotic itself (lanA) as well as for its biosynthesis and modification (*lanBC* or *lanM*), transport (*lanT, lanT+C39 peptidase or lanT+C39 peptidase+lanM*) across the cellular membrane are with a few exceptions encoded on a single gene cluster (Chatterjee et al., 2005; Willey and van der Donk, 2007; Alkhatib et al., 2012; Singh and Sareen, 2014). Furthermore, a two-component system (*lanK* and *lanR*) is present on this operon, which up-regulates the expression of these genes (Kuipers et al., 1995; Qiao et al., 1996).

In order to prevent the harmful effect of the secreted and activated lantibiotic on their own membrane, additional genes (*lanI* and *lanFEG*) encode a lantibiotic specific (auto-)immunity system. Interestingly, although lantibiotics are grouped in different classes based on their size and specific activities (Willey and van der Donk, 2007; Arnison et al., 2013), the lantibiotic specific (auto-)immunity system genes seem to be conserved throughout the species (Alkhatib et al., 2012). The genes of the (auto-)immunity system encode for the following proteins: LanI, a membrane associated lipoprotein, and LanFEG, an ABC transporter localized in the cellular membrane (Draper et al., 2008, 2015). Some lantibiotic strains contain only one of the (auto-)immunity proteins, which correlates with the activity of the produced lantibiotic, which can be membrane binding and/or pore formation activity.

Nisin secreted by some *L. lactis* strains is the most prominent lantibiotic. Due to its high bactericidal activity in combination with low toxicity in humans, nisin has already been used for decades as a natural preservative in the food industry (Delves-Broughton et al., 1996). Active nisin consists of 34 amino acids and contains five stereo-specifically installed lanthionine-based rings. The first three rings (rings A–C) are separated from the last two intertwined rings (rings D–E) by a flexible hinge region (Van de Ven et al., 1991; Zhou et al., 2015). Rings A and B are able to bind lipid II, thereby inhibiting cell growth (Hsu et al., 2004), whereas the hinge region and rings D and E, are able to flip into the membrane (Wiedemann et al., 2001; van Heusden et al., 2002; Hasper et al., 2004). This creates pores, which leads to an immediate eﬄux of nutrients and small compounds, resulting in cell death (Ruhr and Sahl, 1985; Breukink et al., 1999).

**FIGURE 1:**
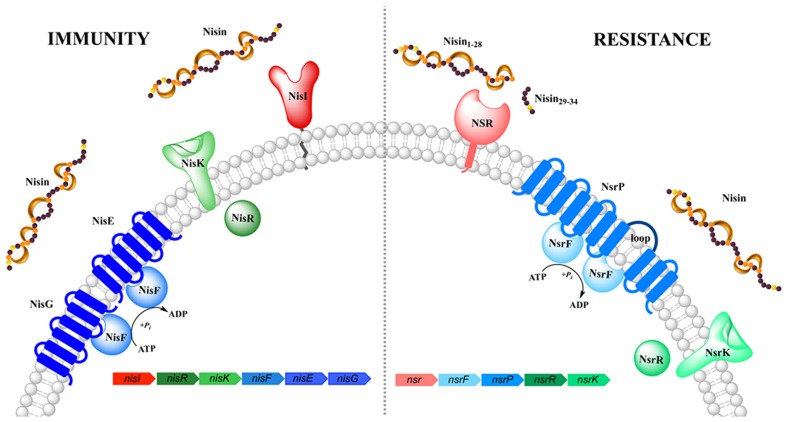
**Schematic representation of the nisin (auto-)immunity and resistance system.** Both systems comprise of a lipoprotein/membrane-associated protein (colored in red/pink, respectively), an ABC transporter (depicted in blue) and a two-component system (shown in green). Functionally similar genes are color coded identically with the exception of NisI and NSR.

The nisin producing *L. lactis* strains are immune against the high antimicrobial activity of nisin via the expression of an distinct immunity system consisting of the lipoprotein NisI and ABC transporter NisFEG (**Figure 1**). When these proteins are expressed, the producing strains survives a high level of immunity against nisin, of up to ~750 nM nisin (1000 IU/ml; Ra et al., 1996). This concentration is a >100-fold higher than the 4–6 nM observed in *L. lactis* strain lacking this immunity system. Interestingly, both lipoprotein and ABC transporter act cooperatively and each of them displays only 10–30% of the full immunity levels when expressed alone (Ra et al., 1999; Stein et al., 2003; AlKhatib et al., 2014a,b).

Such an (auto)-immunity system is conserved and present in al most all lantibiotic producing strain. For example, the subtilin producing *Bacillus subtilis* strain expresses SpaI and SpaFEG (Klein and Entian, 1994); the epidermin producer *Staphylococcus epidermidis* expresses EpiH and EpiFEG (Peschel and Götz, 1996; Otto et al., 1998); the gallidermin producer *Staphylococcus gallinarium* expresses GdmH and GdmFEG and the Pep5 producer *S. epidermidis* expresses PepI (Reis et al., 1994). More examples as well as their operon structure are highlighted in (Alkhatib et al., 2012).

Recently, however, some operons were described encoding a protein defense system against lantibiotics although the host strain is not producing these lantibiotics itself. Interestingly, these operon are mainly found in human pathogenic strains. The expression of the genes localized on these operon results in a resistance against the lantibiotic. For example: *lct*GEF*lcr*XRS in *Streptococcus mutans* conferring resistance against nisin, nukacin ISK-I and lacticin 481 (Kawada-Matsuo et al., 2013), and *gra*XSR/*vra*FG in *S. aureus* provides resistance against various lantibiotics including nisin and nukacin ISK-I (Meehl et al., 2007; Falord et al., 2011, 2012). The *cpr*ABCK-R operon from *Clostridium difficile* confers even resistance against multiple lantibiotics of which nisin, gallidermin, subtilin, and mutacin 1140 (McBride and Sonenshein, 2011; Suárez et al., 2013).

In *Streptococcus agalactiae*, the operon identified confers resistance against the lantibiotic nisin and resembles the genetic architecture of the *nisI* and *nisFEG* immunity genes found in the producing *L. lactis* strain (Khosa et al., 2013; **Figure 1**). The genes encoded on this operon are called *nsr* (encoding for nisin resistance protein) and *nsrFP* (encoding for an ABC transporter), which are probably auto-regulated by a two-component system formed by *nsrR* and *nsrK* (Khosa et al., 2013). The solely expressed membrane-associated nisin resistance protein (also known as NSR) has been shown to confer 20-fold resistance against nisin, when expressed in a sensitive *L. lactis* strain (Khosa et al., 2013). Due to the high sequence homology of NSR, especially the specific TASSAEM region, homologs of this resistance protein have been identified in several other strains such as *S. epidermidis* among others (Khosa et al., 2013).

Recently, the structures of both nisin (auto-)immunity protein NisI from *L. lactis* (Hacker et al., 2015) as well as the resistance protein NSR from *S. agalactiae* (*Sa*NSR; Khosa et al., 2016) have been revealed. They both, although sharing a low sequence similarity of only 23%, protect the cell membrane against the presence of high concentrations of nisin albeit by a different mechanism. Both proteins can be considered as role models for the (auto-)immunity and resistance system of other lantibiotics. Within this review, the function and structure of both proteins will be compared and described.

## The Nisin Immunity Protein NisI

In nisin producing *L. lactis* strains, the LanI immunity protein is called NisI, which is a 27.8 kDa in size comprising of 245 amino acid residues (Kuipers et al., 1993; **Table 1**). The sequence contains a signal peptide which is cleaved off after secretion followed by a site for lipidation (Cys-1 in mature NisI; Kuipers et al., 1993; Sutcliffe and Russell, 1995). The resulting mature NisI (226 amino acid residues and 25.8 kDa in size) is lipid-anchored at the outside of the cytoplasmic membrane (Ra et al., 1996). Approximately, one-third of NisI escapes this lipid modification and is thereby released into the extracellular environment in a lipid-free form that forms an additional mechanism of immunity (Koponen et al., 2004; Takala et al., 2004).

**Table 1:** Characteristics of the nisin immunity protein NisI and nisin resistance protein NSR (* IC_50_ compared against sensitive *Lactococcus lactis* strain NZ9000; Sun et al., 2009; AlKhatib et al., 2014a; Hacker et al., 2015; Khosa et al., 2016).

	NisI	*Sa*NSR
Sequence length	245	320
Molecular weight (kDa)	27.8 (full-length)/25.8 (processed)	36.2
Localization	Membrane attached/lipid-free	Membrane spanning
Sequence motif	N-terminal signal peptide	N-terminal transmembrane helix/conserved TASSAEM region
Function	Nisin binding	Nisin cleavage
Observed mechanism	Reversible cell clustering	Nisin proteolysis
Substrate specificity	N-terminus of nisin: rings A and B	C-terminus of nisin: rings D and E + last six amino acids
Conferred Immunity/resistance *	8–10-fold	18–20-fold
Important residues	Tyr152, Asp155	His98, Ser236
Binding affinity	1 μM	Not determined
Structure determined	NMR	X-ray crystallography
Structure	Mainly β-sheet: two domains and a flexible linker	Helical bundle, protease fold, protease core domain
Binding site determined	NMR/mutational studies	Molecular dynamic simulations/mutational studies

NisI exhibits two functions preventing nisin to reach the cellular membrane. First NisI binds nisin, thereby protecting the nisin-producing bacteria. Important, here is the fact that NisI does not modify or degrade nisin as shown by several independent studies (Qiao et al., 1995; Ra et al., 1996, 1999; Stein et al., 2003; Koponen et al., 2004; AlKhatib et al., 2014a). Initially, the involvement of NisI in immunity was identified via the expression of solely NisI which increased the nisin resistance of both *E. coli* and *L. lactis* (Kuipers et al., 1993). Furthermore, the significance of NisI in the overall nisin immunity of *L. lactis* cells was observed via deletion of the *nisI* gene within the whole nisin operon. The resulting *nisI* knockout strain was more sensitive to nisin than the fully equipped *L. lactis* strain (Siegers and Entian, 1995). Interestingly, the knockout strain of the *nisI* gene had more adverse effects than the knockout of the *nisFEG* genes, leading to the hypothesis that the NisI protein plays a more effective role in the immunity against nisin.

**FIGURE 2:**
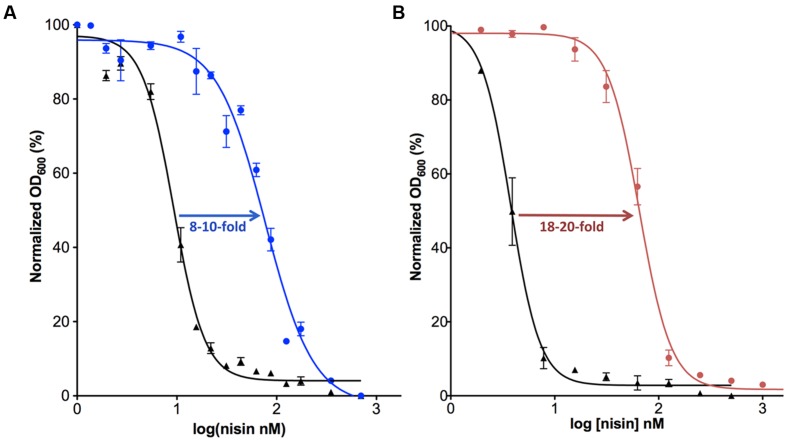
**Activity determination of NisI and *Sa*NSR.** The IC_50_ value was determined for the nisin sensitive *Lactococcus lactis* NZ9000 strain (black) in comparison with the same strain harboring a plasmid containing the *nisI* (**A**; shown in blue) or *nsr* (**B**; shown in pink) gene.

In order to obtain further information on the functioning of the *nisI* gene, it was expressed in a nisin-sensitive *L. lactis* NZ900 strain that contains the *nisRK* two-component system but lacks the rest of the nisin operon and especially the immunity system encoding genes. When NisI was expressed in this strain, 8–10-fold more nisin was needed to inhibit the cell growth by 50% (IC_50_; AlKhatib et al., 2014a; **Figure 2A**). Various studies have shown the importance of the C-terminus of NisI for its activity, especially the last 22 amino acids (Takala and Saris, 2006; AlKhatib et al., 2014a). A deletion of the last 22 residues, reduces the activity of NisI to 30–34% (AlKhatib et al., 2014a). Furthermore, a deletion of only the last five residues decreases the immunity conferred by NisI to approximately 78% (Takala and Saris, 2006). It appears that at low nisin concentration in the environment, the binding capacity of NisI is enough to provide immunity up to a concentration of 60 nM, where the cells are still able to grow. Since the immunity conferred by NisI is a result of its binding to nisin, it is somewhat surprising that NisI *in vitro* displayed a rather weak affinity for nisin as seen by a K_D_ of approximately 0.6–2 μM (Takala et al., 2004; Hacker et al., 2015). However, this is likely due to the fact that the *in vitro* experimental setup lacks the membrane environment.

Recently, in addition to the binding capability of NisI, a second mechanism for conferring immunity by NisI was also described. Upon addition of nisin to *nisI* expressing *L. lactis* cells, the cells start to cluster. Especially at nisin concentrations above the determined IC_50_ value, the cells cluster to form large chains up to a number of 30 cells (AlKhatib et al., 2014a). Importantly, this clustering is only observed when both NisI and nisin are present. Due to this clustering, nisin is unable to reach lipid II. Thereby the activity of nisin, especially the pore forming ability is inhibited as observed via a so-called Sytox assay (AlKhatib et al., 2014a). This clustering mechanism is reversible and as soon as the the external nisin concentration drops below the IC_50_ value, the *L. lactis* cells start growing normally again (AlKhatib et al., 2014a). Thus, when the concentration of nisin increases above a certain threshold (in the reported study around 60–70 nM), which coincides with the measured IC_50_ values, the presence of both NisI and nisin induces a clustering of the *L. lactis* cells. In the nisin sensitive strains lacking *nisI* or upon expressing a *nisI* variant lacking the C-terminally located 22 amino acids, this clustering is not observed, leading to the assumption that the C-terminus of NisI is responsible for this clustering phenomenon (AlKhatib et al., 2014a). This is inline with studies describing that the C-terminus of NisI (last 21 amino acids) interacts with nisin and provides specificity to NisI (Takala and Saris, 2006). Although, this clustering is an interesting observation it has not been unraveled how NisI mediates this.

Since the first two rings of nisin (N-terminal region) are crucial for its binding to lipid II (Wiedemann et al., 2001), which is an initial step in the activity of nisin and also essential for pore formation by nisin, it is a reasonable assumption that NisI recognizes the N-terminus of nisin, presumably the first two rings. Thereby, NisI would directly interfere with the initial and crucial binding event of nisin with lipid II.

## The Nisin Resistance Protein *Sa*NSR

The counter part of NisI in the nisin resistance system of *S. agalactiae* is the nisin resistance protein (*Sa*NSR), which contains 320 amino acids and has a molecular weight of 36.2 kDa (Froseth and McKAY, 1991; Khosa et al., 2013; **Table 1**). *Sa*NSR has a N-terminus hydrophobic region predicted to encode a transmembrane helix (Khosa et al., 2013), thereby *Sa*NSR would be localized at the bacterial membrane (Sun et al., 2009). *Sa*NSR is a nisin degrading enzyme (Sun et al., 2009). Since the lanthionine rings usually cause steric hindrance, thereby inhibiting any protease cleavage a nisin degradation mechanism is quite unique (Wiedemann et al., 2001). *Sa*NSR cleaves off the last six amino acids of nisin, yielding two fragments: nisin^1-28^ and nisin^29-34^. This nisin^1-28^ variant when purified displays 100-fold less bactericidal activity and significantly less affinity toward the bacterial membrane (Sun et al., 2009; AlKhatib et al., 2014a). Thus, the non-producing strains become resistant against nisin by reducing its effectiveness. *Sa*NSR confers 18–20-fold resistance in a nisin sensitive *L. lactis* strain as determined by IC_50_ assays (**Figure 2B**). *Sa*NSR belongs to the S41 family of peptidases and contains a highly conserved TASSAEM sequence motif, which harbors the catalytically active serine residue at position 236 (**Table 1**; Khosa et al., 2013, 2016). This 18–20-fold resistance mediated by *Sa*NSR when expressed in nisin sensitive *L. lactis* cells is almost lost when mutating this Ser236 residue (Khosa et al., 2013).

Furthermore, the resistance mediated by *Sa*NSR in nisin sensitive *L. lactis* strain dropped to mere 1.4–1.7-fold for the nisin variants lacking the rings D and E or only E (Khosa et al., 2016). Additionally, removing the last 6 or 12 amino acids of nisin (nisin^1-28^ and nisin^1-22^, respectively) completely abolished the resistance, clearly indicating the importance of the last ring as well as the C-terminal tail of nisin for recognition by *Sa*NSR. This is in contrast to the specificity of NisI. Nisin shares a high sequence similarity of around 63% with subtilin from *B. subtilis*, and like nisin the lantibiotic subtilomycin also harbors five lanthionine-based rings (Barbosa et al., 2015). Therefore, it might be possible that *Sa*NSR exhibit a broader substrate specificity including resistance toward other lantibiotics as well. Such a multiple lantibiotic resistance specificity has been previously described for an other lantibiotic resistance operon system of *C. difficile* (McBride and Sonenshein, 2011; Suárez et al., 2013).

## Structures of the Immunity Protein NisI and the Resistance Protein NSR

**FIGURE 3:**
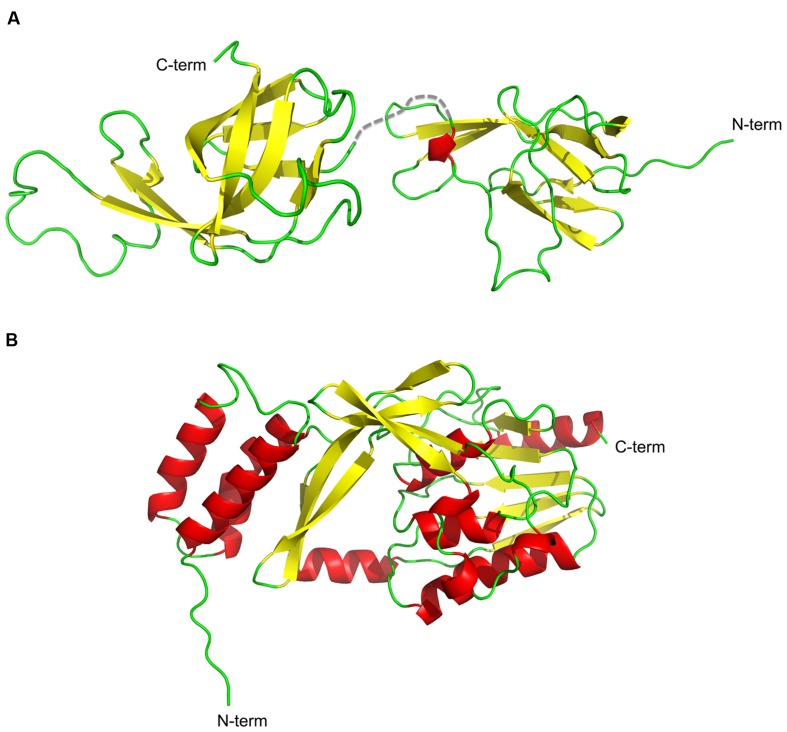
**Cartoon representation of the structures of NisI and *Sa*NSR.** The structures of **(A)** NisI (PDB codes: 2N32 and 2N2E) and **(B)** *Sa*NSR (PDB code: 4Y68) are shown with the secondary elements color coded as red for helices, yellow for sheets and green for the loops. The figure was created with Pymol.

The structure of NisI from *L. lactis* was solved using NMR spectroscopy (Hacker et al., 2015). NisI is a two-domain and predominantly a β-sheet containing protein (**Figure 3A**). The N-terminal part (1–111 residues) is connected to the C-terminal domain (120–226) via a flexible linker (112–119). Interestingly and rather unusual is that both domains adopt a similar unique fold, which has been previously observed for SpaI from *B. subtilis*, the immunity protein against subtilin (Christ et al., 2012). The core of the N- and C- terminal domains of NisI are formed by a seven-stranded antiparallel twisted β-sheet in the strand order β1-β2-β3-β8-β7-β6b-β4a. An extended β-hairpin is formed by strands β4b and β6a that is stabilized by hydrophobic packing interactions with residues from β1 and β2. In addition, the β-hairpin is flanked by a short 3_10_ helix.

**FIGURE 4:**
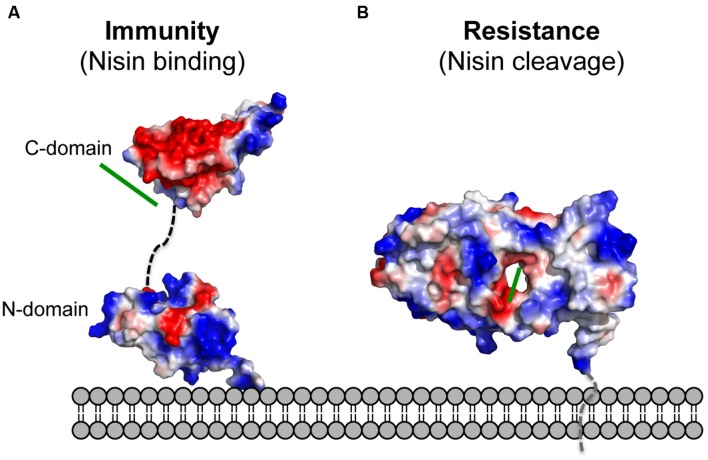
**Electrostatic surface potential of the structures of NisI and *Sa*NSR.** The electrostatic surface potential of **(A)** NisI (PDB codes: 2N32 and 2N2E) and **(B)** *Sa*NSR (PDB code: 4Y68) structures is shown. Negatively charged surface areas are colored in red, while the positively charged areas colored in blue and white areas correspond to hydrophobic surfaces. The determined nisin binding site for both proteins are highlighted with a green line. The figure was created with Pymol.

NisI lacks the N-terminus unstructured region, which is present in SpaI and supposedly allows interaction with the host membrane (Christ et al., 2012; Hacker et al., 2015). Since it was proposed that NisI interacts with the membrane, an elegant set of experiments were performed with the single expressed domains, which revealed that only the N-terminal domain possesses affinity toward the membrane environment (Hacker et al., 2015; **Figure 4**). Therefore, this domain is thought to be localized at the membrane surface. On the contrary, the C-terminal domain of NisI does not bind to lipids. Instead this C-terminal domain binds nisin as observed in NMR titration experiments. This is inline with the previous observations that the C-terminally located last 22 amino acids are important for the functioning of NisI *in vivo.* Although structurally similar, both domains of NisI differ in their surface properties. While the surface of the N-terminal domain of NisI is highly positively charged and interacts with membranes, the C-terminal domain has negatively charged surface with hydrophobic patches and is able to bind nisin (**Figure 4A**), thereby modulating the membrane affinity of the N-terminal domain of NisI by shielding its membrane binding surface (Hacker et al., 2015; **Figure 4A**). NisI has not been shown to interact with other lantibiotics and displays rather high substrate specificity. A protein consisting of the N-terminal SpaI domain fused to the C-terminal domain of NisI, however, displays immunity against nisin (Takala and Saris, 2006). This highlights that the nisin binding site is localized within the C-terminus of NisI. Since subtilin and nisin share high homology in terms of sequence as well as lanthioinine ring positions it is not surprising that the domain structure of NisI and SpaI are structurally similar. This in contrast to the structure MlbQ from the actinomycete *Microbispora* ATCC PTA-5024 conferring resistance against NAI-107 which was solved by NMR (Pozzi et al., 2015). No significant sequence identity as well as structural similarity was observed between NisI and MlbQ. This likely is due to the larger difference between nisin and NAI-107 in sequence as well as lanthionine ring composition. It seems that the LanI immunity proteins evolved differently and are highly specific for their cognate lantibiotic.

The crystal structure of nisin resistance protein from *S. agalactiae* (*Sa*NSR; without the N-terminal transmembrane helix) was solved using X-ray crystallography and consists of eleven α-helices and eleven β-strands (**Figure 3B**; Khosa et al., 2016). *Sa*NSR is composed of three domains: an N-terminal helical bundle comprising of 65 amino acid residues (Lys31-Gly96), form helices α1-α3. This domain ends in a triple glycine motif before entering the protease cap domain. This protease cap domain consists of helix α4 and a β-hairpin structure formed by two strands and forms a lid-like structure above the tunnel. The last domain called the protease core is formed by six strands βb-βg and five helices α5-α9 and adopts a ‘protease fold’ domain as observed in other S41 peptidases (Liao et al., 2000; Kim et al., 2002; Mastny et al., 2013). Together they form a hydrophobic tunnel of ~10 Å width. This hydrophobic, negatively charged tunnel is responsible for binding to nisin by ‘roping in’ the peptide (**Figure 4B**). The protease core domain also contains the highly conserved TASSAEM region that harbors the previously identified catalytically active serine at position 236 (Khosa et al., 2013).

The active center of *Sa*NSR consists of a catalytic dyad formed by residues Ser236 (Khosa et al., 2013), which is part of the TASSAEM motif, and His98 as determined by mutational analysis (Khosa et al., 2016) and also described for some other proteases (Page and Di Cera, 2008).

Unfortunately, the crystal structure lacks the substrate nisin. Instead a peptide called N-pep, belonging to symmetry-related molecule was bound within the tunnel. This information was used for molecular dynamic simulation studies to determine the nisin-binding site. It was observed that nisin is stably bound in the tunnel formed in between the domains of *Sa*NSR. Additionally, the residues forming the hydrophobic interactions for proper orientation of rings D and E of nisin are embedded in the protease core domain within or on the outside of the tunnel that is situated in the middle of *Sa*NSR protein.

The model of *Sa*NSR/nisin complex demonstrates the significance of C-terminally located lanthionine rings D and E of nisin for substrate specificity (Wiedemann et al., 2001). The importance of these rings was also highlighted by mutational analyses of nisin. Here, *Sa*NSR did not recognize nisin variants lacking the last or the last two rings.

## Conclusion

The expression of NisI and NSR reduce the activity of nisin. In the nisin producing strains, the immunity protein NisI solely binds nisin thereby reducing the amount of nisin reaching the lipid II target molecule in the membrane. To achieve this NisI not only binds to nisin but also induces a “shielding mechanism” of the *L. lactis* cells. Immunity is thereby provided without harming or modifying the own lantibiotic produced.

In contrast, NSR present in nisin non-producing strains cleaves nisin in two parts, thus, reducing the ability of nisin to form pores in the membrane.

NisI and NSR, however, represent the first line of defense and within both immunity and the resistance operons, an additional protein system is also encoded. In case of the immunity system, it is the ABC transporter NisFEG and for the resistance system, it is NsrFP. It has been shown that NisFEG and NisI act cooperatively. However, for the nsr system such a cooperative mechanism has not been detected so far and it has to be further investigated whether these resistance proteins are also able to act together in their battle against the lantibiotic nisin. Although, lantibiotics are considered to be powerfull biological antimicrobial compounds already resistance mechanisms are present in some human pathogenic strains. This hampers the usage of lantibiotics for medical purposes. A detailed knowledge about these resistance mechanisms would pave a way to bypass these inherently present systems.

## Author Contributions

SK, ML, and SS wrote the manuscript. SK, ML prepared the figures. SK, ML, and SS finalized the manuscript.

## Conflict of Interest Statement

The authors declare that the research was conducted in the absence of any commercial or financial relationships that could be construed as a potential conflict of interest.
